# ICTV Virus Taxonomy Profile: *Wupedeviridae* 2023

**DOI:** 10.1099/jgv.0.001932

**Published:** 2023-12-20

**Authors:** Jens H. Kuhn, Scott Adkins, Katherine Brown, Juan Carlos de la Torre, Michele Digiaro, Holly R. Hughes, Sandra Junglen, Amy J. Lambert, Piet Maes, Marco Marklewitz, Gustavo Palacios, Takahide Sasaya, Massimo Turina, Yong-Zhen Zhang

**Affiliations:** ^1^​ Integrated Research Facility at Fort Detrick, National Institute of Allergy and Infectious Diseases, National Institutes of Health, Fort Detrick, Frederick MD 21702, USA; ^2^​ United States Department of Agriculture, Agricultural Research Service, US Horticultural Research Laboratory, Fort Pierce, FL 34945, USA; ^3^​ Division of Virology, Department of Pathology, Addenbrookes Hospital, University of Cambridge, Cambridge CB2 0QN, UK; ^4^​ Department of Immunology and Microbiology IMM-6, The Scripps Research Institute, La Jolla CA 92037, USA; ^5^​ CIHEAM, Istituto Agronomico Mediterraneo di Bari, 70010 Valenzano, Italy; ^6^​ Centers for Disease Control and Prevention, Fort Collins CO 80521, USA; ^7^​ Institute of Virology, Charité-Universitätsmedizin Berlin, Corporate Member of Freie Universität Berlin, Humboldt-Universität zu Berlin, and Berlin Institute of Health, Berlin 10117, Germany; ^8^​ KU Leuven, Rega Institute, Zoonotic Infectious Diseases Unit, 3000 Leuven, Belgium; ^9^​ FIND, 1202 Geneva, Switzerland; ^10^​ Department of Microbiology, Icahn School of Medicine at Mount Sinai, New York, NY 10029, USA; ^11^​ Institute for Plant Protection, National Agriculture and Food Research Organization, Tsukuba, Ibaraki 305-8517, Japan; ^12^​ Institute for Sustainable Plant Protection, National Research Council of Italy (IPSP-CNR), 10135 Torino, Italy; ^13^​ School of Life Sciences and Human Phenome Institute, Fudan University, Shanghai 201052, PR China

**Keywords:** ICTV Report, taxonomy, *Wupedeviridae*, wumivirus, Wǔhàn millipede virus 2

## Abstract

*Wupedeviridae* is a family of negative-sense RNA viruses with genomes of about 20.5 kb that have been found in myriapods. The wupedevirid genome consists of three monocistronic RNA segments with open reading frames (ORFs) that encode a nucleoprotein (NP), a glycoprotein (GP), and a large (L) protein containing an RNA-directed RNA polymerase (RdRP) domain. This is a summary of the International Committee on Taxonomy of Viruses (ICTV) Report on the family *Wupedeviridae*, which is available at ictv.global/report/wupedeviridae.

## Virion

Unknown.

## Genome

**Fig. 1.**
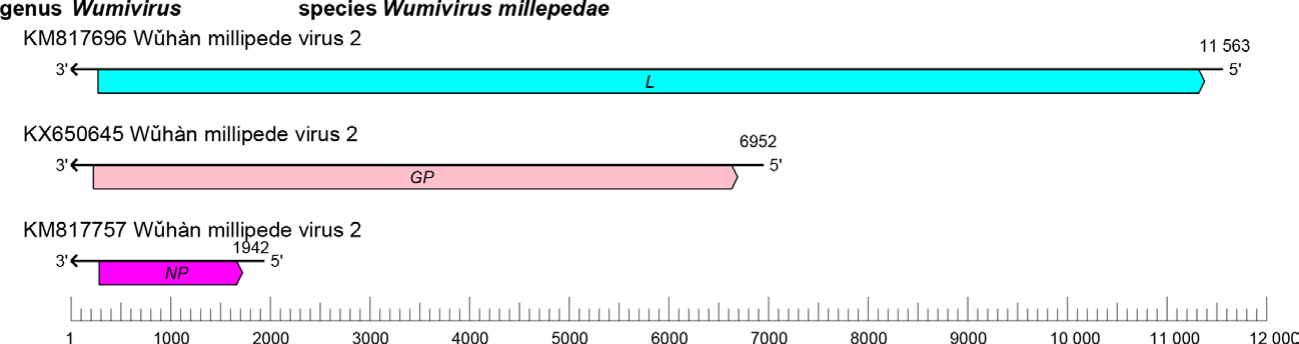
Genome organisation of Wǔhàn millipede virus 2. ORFs are coloured according to the predicted protein function (*GP*, glycoprotein gene; *L*, large protein gene; *NP*, nucleoprotein gene).

The wupedevirid genome comprises three RNA segments (small [S], medium [M], and large [L]) of linear negative-sense RNA with a total length of about 20.5 kb (S segment: about 1.9 kb; M segment: about 7.0 kb; and L segment: about 11.6 kb) (Table 1). Each segment contains an ORF that encodes an NP (S segment), a GP (M segment), and an L protein containing an RdRP domain (L segment) [1] (Fig. 1).

**Table 1.** Characteristics of members of the family *Wupedeviridae*

Example	Wǔhàn millipede virus 2 (S: KM817757; M: KX650645; L: KM817696), species *Wumivirus millepedae*, genus *Wumivirus*
Virion	Unknown
Genome	About 20.5 kb of trisegmented negative-sense RNA
Replication	Unknown
Translation	Unknown
Host range	Polydesmid myriapods (millipedes)
Taxonomy	Realm *Riboviria*, kingdom *Orthornavirae*, phylum *Negarnaviricota*, class *Ellioviricetes*, order *Bunyavirales*; the family includes the genus *Wumivirus* and the species *Wumivirus millepedae*

## Replication

Unknown.

## Taxonomy

**Fig. 2.**
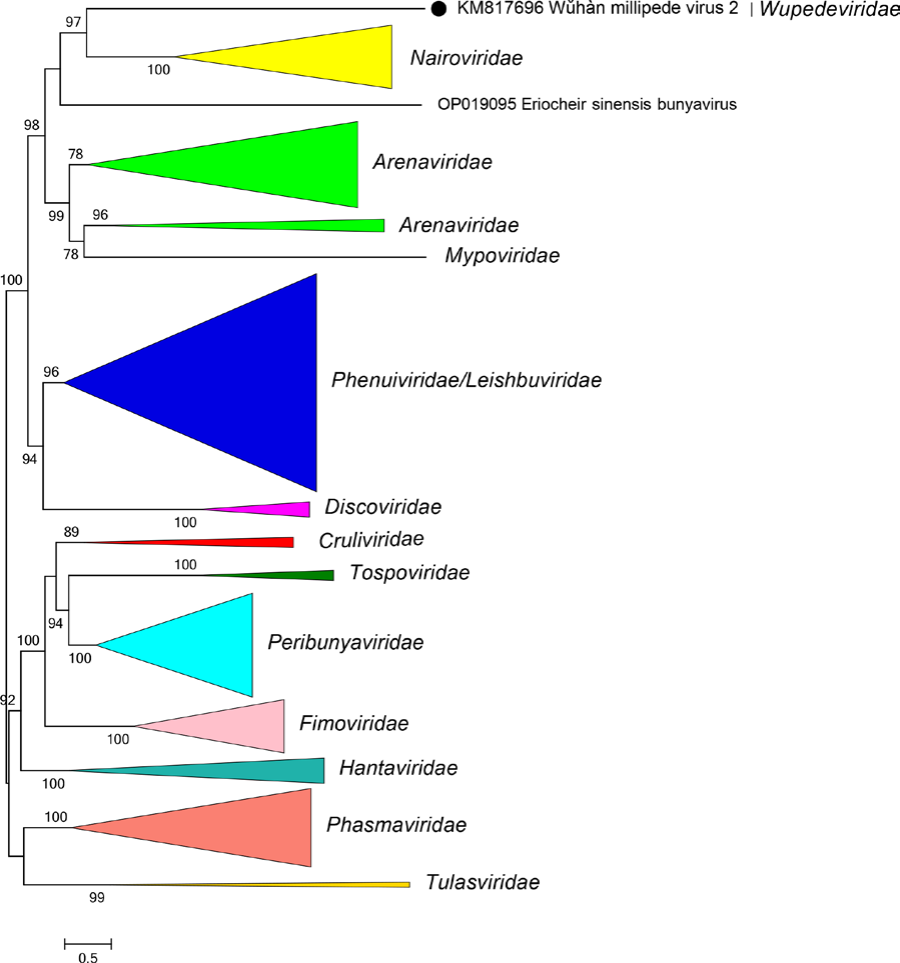
Phylogenetic relationships of Wǔhàn millipede virus 2. Family branches have been collapsed. Numbers at nodes indicate bootstrap support >70 %. For details of viruses and methods see the full ICTV Report on the family *Wupedeviridae*.

Current taxonomy: ictv.global/taxonomy. Wupedevirids are most closely related to arenavirids, discovirids, leishbuvirids, mypovirids, nairovirids, and phenuivirids [2–4] (Fig. 2). The family includes the genus *Wumivirus* for viruses that infect myriapods (millipedes). Like most closely related viruses [3, 5], wupedevirids (i) have multisegmented, negative-sense single-stranded RNA genomes; (ii) encode proteins with high sequence identity to proteins of other bunyavirals; (iii) and have five conserved motifs (A–E) in their RdRP domain.

## Resources

Full ICTV Report on the family *Wupedeviridae*: ictv.global/report/wupedeviridae.
